# Persistence of Matrilocal Postmarital Residence Across Multiple Generations in Southern Africa

**DOI:** 10.1007/s12110-023-09452-4

**Published:** 2023-06-13

**Authors:** Austin W. Reynolds, Mark N. Grote, Justin W. Myrick, Dana R. Al-Hindi, Rebecca L. Siford, Mira Mastoras, Marlo Möller, Brenna M. Henn

**Affiliations:** 1 Department of Anthropology, Baylor University, Waco, TX USA; 2 Department of Anthropology, University of California (UC), Davis, CA USA; 3 School of Human Evolution and Social Change, Arizona State University, Tempe, AZ USA; 4 Department of Biomolecular Engineering, University of California, Santa Cruz, CA USA; 5 UC Davis Genome Center, Davis, CA USA; 6 DSI-NRF Centre of Excellence for Biomedical Tuberculosis Research, South African Medical Research Council Centre for Tuberculosis Research, Division of Molecular Biology and Human Genetics, Faculty of Medicine and Health Sciences, Stellenbosch University, Cape Town, South Africa

**Keywords:** Southern Africa, Khoe-San, Postmarital residence patterns, Colonialism, Cultural persistence, Cultural evolution

## Abstract

Factors such as subsistence turnover, warfare, or interaction between different groups can be major sources of cultural change in human populations. Global demographic shifts such as the transition to agriculture during the Neolithic and more recently the urbanization and globalization of the twentieth century have been major catalysts for cultural change. Here, we test whether cultural traits such as patri/matrilocality and postmarital migration persist in the face of social upheaval and gene flow during the past 150 years in postcolonial South Africa. The recent history of South Africa has seen major demographic shifts that resulted in the displacement and forced sedentism of indigenous Khoekhoe and San populations. During the expansion of the colonial frontier, the Khoe-San admixed with European colonists and enslaved individuals from West/Central Africa, Indonesia, and South Asia, introducing novel cultural norms. We conducted demographic interviews among Nama and Cederberg communities representing nearly 3,000 individuals across three generations. Despite the history of colonial expansion, and the subsequent incorporation of Khoe-San and Khoe-San-descendant communities into a colonial society with strong patrilocal norms, patrilocality is the least common postmarital residence pattern in our study populations today. Our results suggest that more recent forces of integration into the market economy are likely the primary drivers of change in the cultural traits examined in our study. Birthplace had a strong effect on an individual’s odds of migration, distance moved, and postmarital residence form. These effects are at least partially explained by the population size of the birthplace. Our results suggest that market factors local to birthplaces are important drivers of residence decisions, although the frequency of matrilocal residence and a geographic and temporal cline in migration and residence patterns also indicate the persistence of some historic Khoe-San cultural traits in contemporary groups.

**Supplementary Information:**

The online version contains supplementary material available at 10.1007/s12110-023-09452-4.

Over the past 400 years, European colonization has resulted in a major decline in global cultural diversity. Under the rule of colonial governments, indigenous groups around the world were forced to assimilate into colonial culture. Through this process, indigenous groups changed or lost primary languages, settlement patterns and marriage preferences, burial practices, foodways, and other cultural traits (Bacon, 2019; Fitzhugh, 1985; Khawaja, 2021; Murray, 2004; Silliman, 2005). More recently, industrial modernization and globalization have acted as homogenizing forces, further putting indigenous lifeways at risk of extinction (Allen, 2006; Jovel et al., 2009). Although the forces of colonialism and globalization are the most recent and widespread, cultural change as a result of interaction between groups has happened for millennia. For example, Indo-European languages are thought to have spread with expanding pastoralist populations during the Bronze Age and have become the dominant language family of Europe (Bouckaert et al., 2012; Fortunato & Jordan, 2010). Patrilocal residence patterns also spread widely across sub-Saharan Africa as Bantu-speaking groups moved to the east and south (Moravec et al., 2018; Opie et al., 2014).

But these cultural shifts have not universally resulted in culture loss in indigenous communities. Recently, scholarship has focused on the resilience of some cultural traits despite strong external influences (Giuliano & Nunn, 2021; Hofman et al., 2020; Panich, 2013). In this study, we use multigenerational data on the migration and postmarital residence patterns of two Khoe-San descendant populations in South Africa to examine the dynamics of these cultural traits over the past 150 years. Despite such influences as patrilocal colonialism, industrialization, apartheid governance, and current secular trends, we find considerable variation in locality patterns, including the persistence of matrilocality.

Human cultural rules and norms of descent vary widely, though they commonly follow rules tracking consanguineal kinship. However, postmarital residence patterns often differ from kinship and inheritance patterns. Cultural incest taboos and incest avoidance psychology invariably create a situation in which at least one marriage partner will live among affinal rather than consanguineal kin (Murdock, 1949). Postmarital residence patterns—the residence forms pertaining to the settlement of newly married couples—are the primary set of kinship norms regulating human dispersal (Levinson & Malone, 1980). Patrilocal residence, where the wife lives with or near the husband’s kin, is the most common pattern worldwide, occurring in approximately 70% of societies (Levinson & Malone, 1980). Other patterns include matrilocality, where the husband lives with or near the wife’s kin, and ambilocality, where the couple may choose to live with either set of kin (Holy, 1996). Cross-cultural comparative analyses have shown that matrilocality is quite common among foraging societies worldwide for a first birth, followed by multilocality for subsequent births—a pattern that is seen more in forager groups than in groups with other forms of subsistence (Marlowe, 2004) and often co-occurs with matrilineal descent and inheritance patterns (Surowiec et al., 2019). Matriliny and matrilocality have also been shown to be negatively correlated with presence of movable property such as herds (Ember & Ember, 1971; Murdock, 1949). A growing body of research in quantitative human ecology is focused on reconstructing the ancestral kinship norms of human populations and how they have changed through prehistory (Fortunato et al., 2006; Holden & Mace, 2003; Jordan et al., 2009; Opie et al., 2014). A recent study found that among more than 1200 populations in the D-PLACE ethnographic atlas (Kirby et al., 2016), large community size, hierarchy beyond the local level, and intensive agriculture were all negatively associated with matrilocality, whereas foraging modes of subsistence were positively associated with matrilocality (Surowiec et al., 2019).

The history of South Africa provides a rich context for studying how migration and settlement norms have responded to the pressures of colonialism and globalization. The indigenous populations of South Africa, collectively referred to as Khoe-San, have been a focus of anthropological and genetic research because of their traditional subsistence patterns (predominately foraging), unique linguistic families—all of which contain click consonants—and their deep genetic divergence time from other human groups (Barnard, 1992; Henn et al., 2011). Historic and ethnographic evidence from many Khoekhoe and San groups have reported a preference for either matrilocal or ambilocal postmarital residence (Barnard, 1992; Heinz, 1994; Lee, 1984; Silberbauer, 1981; Widlok, 1999). In contrast, historical European populations have been patrilocal (Blaauboer & Mulder, 2010; Fortunato & Jordan, 2010).

As the colonial frontier in South Africa expanded from Cape Town, the descendants of enslaved individuals and servants whose parentage was derived from multiple ancestries emerged (Penn, 2005). Their social standing in colonial society varied, but as the eighteenth century progressed, discrimination against these communities increased, resulting in many individuals fleeing to the northern and eastern frontiers of the Cape Colony. As these communities dispersed from the expanding colony, they encountered various indigenous Khoe-San groups and, in many cases, became integrated with them (Penn, 2005) or formed new communities, such as the Oorlam, Griqua, or “Baasters” (Edgar & Saunders, 1982; Nurse & Jenkins, 1975; Ross, 1975). Today these communities in the Northern and Western Cape Provinces are often referred to as “Coloured,” an artifact of the postcolonial and apartheid South African terminology (Adhikari, 1992, 1996). Although the ethnic term “Coloured” already consisted of several sub-categories, following the Population Registration Act of 1950, the term became solidified as a macro-classification, lumping together many multi-ethnic communities across South Africa, as well as Khoe-San, Malay, and Indian communities (Oakley, 2006).

**Fig. 1:**
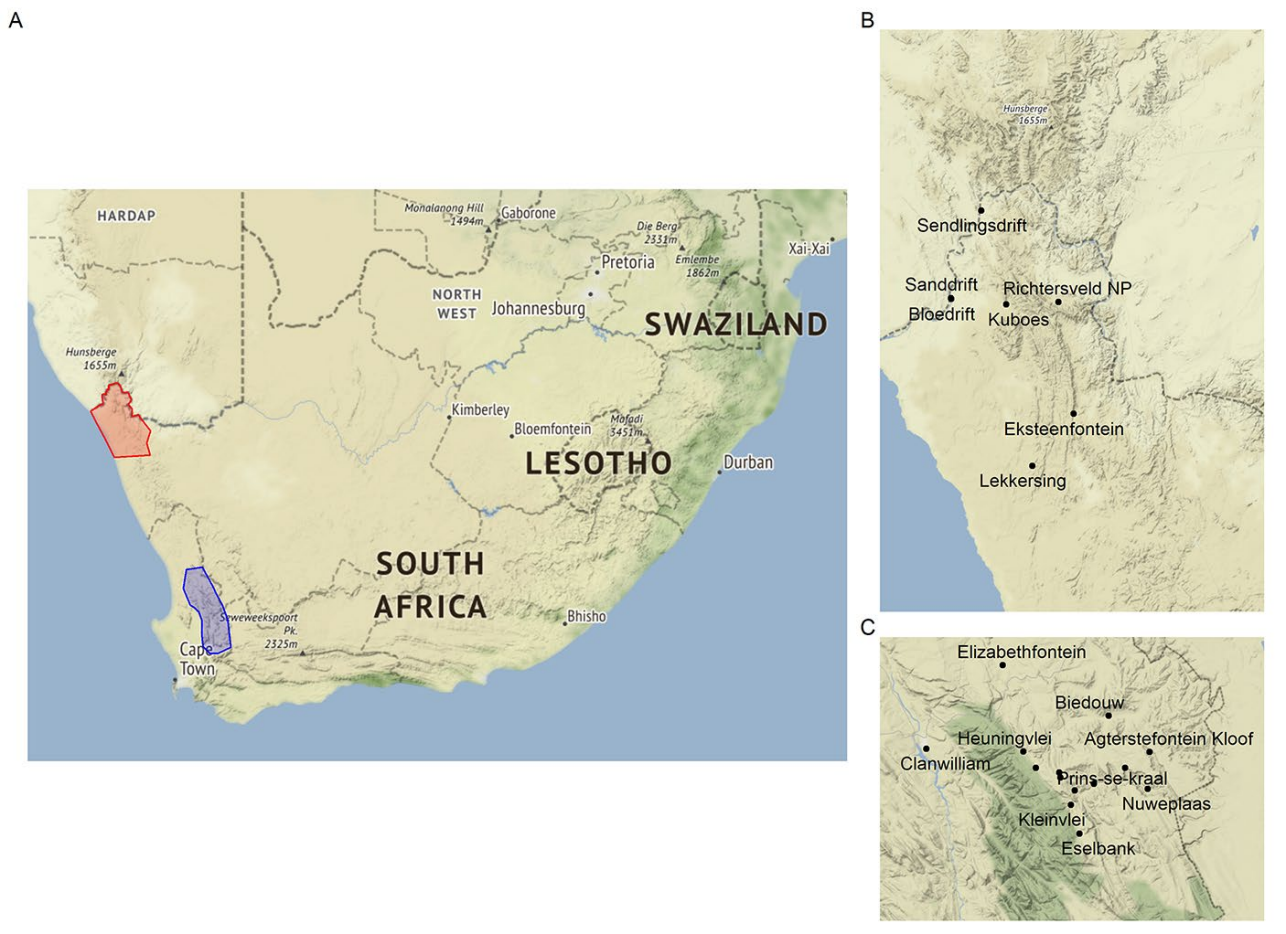
Map of the study regions (A) with the Richtersveld highlighted in red and the Cederberg highlighted in blue, and sampling locations in the Richtersveld (B) and the Cederberg (C)

In this study, we examine migration and residence patterns of three generations of rural South Africans from these Khoe-San descendant groups to test whether cultural indicators of Khoe-San origins are maintained in these communities today. We sampled a collection of Coloured communities in the Cederberg Mountains (Western Cape Province) and the Nama of the Richtersveld (Northern Cape Province) (Fig. 1). The Cederberg is a mountainous region north of Cape Town made up primarily of small towns and farmsteads, bordered by the arid Karoo dry steppe to the east and semi-desert Namaqualand to the north. Because of the difficult topography, the Cederberg was one of the first natural barriers to colonial expansion, serving as a natural refuge for Khoe-San as well as individuals fleeing the spread of the colonial frontier until the late eighteenth century, when it became fully incorporated into the Cape Colony (Mitchell, 2002; Penn, 2005). During the precolonial period, the Cederberg Mountains were home to both foraging populations and Khoekhoe pastoralists. The majority of study participants from this region today self-identify as Coloured and work as subsistence farmers. Moving further from Cape Town, the Richtersveld is an arid desert landscape in the northwest corner of South Africa, bordered to the north by the Orange River and Namibia, and to the west by the Atlantic Ocean. Permanent European settlement did not begin in this region until the early nineteenth century (Rohde & Hoffman, 2008). Study participants in the Richtersveld primarily self-identify as Nama, a group of Khoekhoe pastoralists who have lived in this region for hundreds of years (Smith, 1995). Some still maintain traditional subsistence patterns of sheep and goat herding, alongside wage labor at local diamond mines.

Because of the history of colonial expansion in South Africa, and the subsequent incorporation of Khoe-San and Khoe-San-descendant communities into white South African society, our expectation was to see the adoption of Dutch and British colonial cultural traits in our study populations, such as patrilocal residence and associated sex-biased migration patterns, and a corresponding decrease in matrilocality. Because it is much closer to the colonial center of Cape Town and was exposed to European colonists nearly 100 years earlier, we hypothesized that there would be less matrilocal residence in the Cederberg than in the Richtersveld. However, our results suggest that more recent forces, such as acculturation, apartheid policies related to migration, urbanization, and integration into the global market economy (variably referred to as modernization and/or globalization) are likely the primary drivers of change in the cultural traits in our period of study.

To learn more about past interactions between Khoe-San groups and European colonizers, we incorporated genetic data into our analysis. Specifically, we examined admixture patterns (i.e., individual genetic ancestry from recent Khoe-San, European, and/or Bantu-speaking African ancestors) as a crude proxy for past cultural interaction. We hypothesized that the more recent ancestry a participant had from European sources, the more cultural interaction there would have been between that person’s Khoe-San and European ancestors. Similar analyses have been done with other cultural traits. For example, Verdu et al. (2017) found that genetic admixture and language changes followed similar patterns in Cape Verdean Kriolu speakers, with possible co-transmission of genetic and linguistic variation. However, we find that genetic ancestry does not provide any additional explanatory power for the migration and locality patterns we examined in this study.

## Data and Methods

### Sample Collection and Ethics Approval

Demographic and genetic data were collected from 411 individuals from two rural regions of the Northern and Western Cape Provinces of South Africa. Sampling of 204 individuals in the Cederberg region took place in 2017. Sampling of 207 individuals in the Richtersveld region took place in 2014 and 2015. Local community members, local community leaders, traditional leaders, nonprofit organizations, and a legal counselor were all consulted regarding the aims of the research before collection of DNA. Institutional review board (IRB) approval was obtained from Stony Brook University (727494-1), University of California at Davis (1220180-7), and Stellenbosch University (N11/07/210). All individuals gave signed written and verbal consent with a witness present before participating.

### Demographic Data

Ethnographic interviews were conducted, including questions about age, language, place of birth, and self-identified ethnic group of the individual and of his/her mother, maternal grandparents, father, and paternal grandparents. We recorded the familial relationships between any sampled individuals, if revealed during the interview, and removed siblings at random from our analysis to avoid double counts in our demographic information. After this removal, we were left with 391 individuals (*N* = 195 Cederberg; *N* = 196 Richtersveld).

Our capture of demographic data depended on informants’ knowledge and recall of life-history information of their forebears. For example, our ability to record migrations of parents or grandparents was limited by the extent to which an informant could name these individuals’ birthplaces, and in a number of cases the informant had only incomplete information. We report on and discuss our treatment of various forms of censoring and missingness in these data in subsequent sections.

Distances between the birthplaces of an individual and those of their parents were obtained from a pairwise distance matrix for all birthplaces in the sample (proband and mother, proband and father, mother and maternal grandmother, mother and maternal grandfather, father and paternal grandmother, father and paternal grandfather). Migration distances were measured between birthplaces using Haversine great-circle distances as implemented in the *geosphere* v1.5-10 R package (Hijmans et al., 2019). If an individual’s birthplace was known only at the regional level, such as “in the Richtersveld,” a regional polygon was used to calculate a maximum and minimum possible distance between the individual’s birthplace and those of their parents and/or offspring. Cases such as these, in which migration distances are not known exactly but can be bounded above and below, produce interval censored outcomes (see, e.g., Little & Rubin 2002, Sect. 15.3). They do contain information about migration distances—in a coarsened form—and have been incorporated in our statistical analyses using methods described in subsequent sections.

Postmarital residence patterns were inferred by comparing the birthplace of an individual and the birthplaces of both their mother and father (see Figure S3 in the Electronic Supplemental Material [ESM]). Because of the nature of our data, we define locality patterns based on shared birthplaces between parents and children. If the individual was born in the same place as both parents, we recorded the pattern as equilocal. If an individual was born in the same location as their mother but a different location than their father, we recorded the pattern as matrilocal, and so on. For each family in our dataset, three measurements of postmarital residence were recorded: one comparing participants’ birthplaces with those of their mother and father, one comparing a participant’s mother’s birthplace to that of the mother’s mother and mother’s father, and one comparing a participant’s father’s birthplace to that of the father’s mother and father’s father. This differs from the way these terms are more commonly used in the literature, which typically defines locality pattern based on where a newly married couple chooses to take up residence (e.g., uxorilocality when the newly married couple takes up residence in the household of the bride’s parents or virilocality when they live in the household of the groom’s parents).

### Genetic Data

DNA samples were collected from saliva with Oragene OGR-500 kits (DNA Genotek) and extracted using prep-IT L2P reagents (DNA Genotek) according to the manufacturer’s protocol. All samples were genotyped for > 2 million single nucleotide polymorphisms (SNPs) on the Illumina H3Africa array. Raw genotype data was processed with Illumina’s GenomeStudio to call common variants (MAF > 0.05), followed by zCall to call rare variants (Goldstein et al., 2012). Bioinformatic pipelines are publicly available via github (https://github.com/hennlab/snake-SNP_QC). The genotyped data was further cleaned using plink2 (Chang et al., 2015) with the following parameters: --mind 0.1 --geno 0.05 to remove individuals missing a large number of SNPs and SNPs missing in a large number of study participants, respectively.

### Genetic Ancestry Estimation

For admixture, the following additional QC flags were applied: --hwe 0.001 --maf 0.01 --indep-pairwise 200 25 0.4. 648 individuals from 8 populations were used as reference groups. Admixture estimates were calculated using ADMIXTURE v1.13 (Alexander et al., 2009). This was done in groups of maximally unrelated individuals to avoid biasing the ancestry estimates. ADMIXTURE was run for *k* = 3 to *k* = 6 on unsupervised mode for each of the groups. After matching clusters, we merged ancestry estimates across all groups, averaging individuals that appeared in multiple running groups using pong (Behr et al., 2016). We focus on *k* = 3 results in our models as this captures the Khoe-San, West African, and Eurasian ancestry clusters of interest; additional ancestry partitions are possible but would greatly increase the complexity of the models.

### Modeling Intergenerational Migration

Migration, as measured here, consists of two linked outcomes: whether or not an individual migrated, and if so, how far. We model these outcomes jointly using a two-part model (sometimes called a *hurdle* model; Cragg 1971) in which the first part gives the probability of migration and the second part, the distance migrated, if migration occurred. The probability density for a migration distance $d\ge 0$ then has the form

$$h\left(d;{\pi },\varvec{\gamma }\right)=\left\{\begin{array}{c}1-\pi;d=0\\ \pi g\left(d;\varvec{\gamma }\right);d>0\end{array}\right.$$

where *π* is the probability of migration and *g* is a suitable probability density for a non-negative variable having parameter *γ*. This two-part formula has diverse applications, for example in modeling human foraging returns (McElreath & Koster, 2014) and traffic crash rates (Ma et al., 2015).

The two parts of *h* contain detailed sub-models used here to capture hierarchical variation in migration. For individual *i*, belonging to extended family *l(i)* and having birthplace *b(i)*, the log-odds of migration are

$$\text{l}\text{o}\text{g}\text{i}\text{t}\left({{\pi }}_{\text{i}}\right)={\varvec{x}}_{\varvec{i}}^{\varvec{{\prime }}}\varvec{\beta }+{S}_{l\left(i\right)}+{T}_{b\left(i\right)}$$

and the natural logarithm of the distance migrated, given that the individual migrated, is

$$\text{log}\left({d}_{i}\right)={\varvec{x}}_{\varvec{i}}^{\varvec{{\prime }}}\varvec{\gamma }+{U}_{l\left(i\right)}+{V}_{b\left(i\right)}+{E}_{i}$$

where ***x***_***i***_ is a vector of covariates for individual *i* (here, the same covariates for both sub-models); **β** and *γ* are vectors of regression parameters; *S* and *U* are family-level varying intercepts for migration and distance migrated, respectively; *T* and *V* are analogous birthplace-level varying intercepts; and *E* is an error term completing a log-normal specification for the probability density *g* in the second part of *h*.

The covariates ***x***_***i***_ in our baseline model include an additive term for the *subject’s birth year*, used to adjust for secular trends in migration; additive terms for *sex*, *generation* (subject’s *parents* or *grandparents*) and *region* (Cederberg or Richtersveld); along with pairwise and three-way interactions of *sex, generation* and *region*. The full-factorial model for *sex, generation*, and *region* allows each of the six combinations of these variables to have a unique effect on migration. Elaborations of the baseline model include either additive terms for the *size* of an individual’s birthplace (coded as a categorical variable for *city*, *town*, *farm*, or *unknown*) or additive terms for the subject’s *genetic ancestry* (inferred proportion of Khoe-San ancestry relative to Eurasian ancestry, and similarly for West African ancestry relative to Eurasian).

The hierarchical structure of the sample, featuring the clustering of individuals within families and, separately, within shared birthplaces, makes the inclusion of varying intercepts *S, T, U*, and *V* in the migration model both an obligation and an opportunity. These terms are sometimes called random effects (Searle et al., 1992). Here, varying intercepts are included to adjust for non-independence of migration decisions within family lineages and among individuals sharing the same birthplace, as well as to incorporate contextual effects of families and birthplaces that may not be captured by measured covariates (Jones, 1991; Merlo et al., 2005).

Finally, the model for migration distance must accommodate two forms of *censoring*. The first occurs, for example, when a parent and child were born in the same region, yet the parent’s exact birthplace is not known. Here, the parent’s migration distance can be bounded above by the distance between the child’s birthplace and the farthest location from it within the shared natal region. The second form of censoring occurs when a parent and child were born in different regions, and the parent’s exact birthplace is again unknown. Here, the parent’s migration distance can be similarly bounded above, and further bounded below by the distance between the child’s birthplace and the *nearest* location to it within the parent’s natal region. The computational method described below incorporates these forms of censoring appropriately.

For 629 of 2346 parent-child combinations in the sample, no information about migration was available: these present a loss of information beyond censoring because the two linked outcomes (whether or not an individual migrated, and how far if they did) are entirely missing. Imputation strategies are used fairly routinely when missingness is prominent in a sample (Little & Rubin, 2002), though application of such methods is most straightforward when missingness occurs in the covariates. Here, the covariates are fully observed, and the main concern is the extent to which missingness might be related to the unobserved outcomes or secondary consequences of them (Little & Rubin, 2002). Broadly, imputation of missing outcomes would require that we posit a statistical model predicting the occurrence of missingness along with the unobserved outcomes, which we are reluctant to do in the absence of independent theory and information. For all practical purposes, our analyses incorporate only cases for which outcomes were at least partially observed.

### Modeling Locality Patterns

Postmarital residence in our sample is a categorical outcome from the set ***R*** = {*equilocal, neolocal, matrilocal, patrilocal*} for each married couple. One category in ***R*** is necessarily redundant, as, for example, if a couple’s residence was neither neolocal, matrilocal, nor patrilocal, it must have been equilocal. The *multi-logit* model used here acknowledges this redundancy by comparing frequencies of three of the categories in ***R*** to an arbitrary reference category. Different choices for the reference category produce algebraically equivalent models and therefore equivalent inferences (Agresti, 1990). We use the most common residence type in our sample, *equilocality*, as the reference category. The model then captures hierarchical variation in the log odds of each residence type *r* (among neolocal, matrilocal or patrilocal), relative to equilocal residence. Mberu (Mberu, 2005) used a multi-logit model to investigate the relationship between migration decisions and social and demographic covariates in rural Nigeria. Koster and McElreath (2017) analyzed a “spot check” human time allocation data set with a series of increasingly complex multi-logit models and gave useful conceptual and computational details.

As for the migration model, we use varying intercepts in the postmarital residence model to adjust for unique effects of family lineages and birthplaces. For married couple *c*, belonging to extended family *l(c)*, and in which the male’s and female’s birthplaces were respectively *b*_*m*_*(c)* and *b*_*f*_*(c)*, the log odds of residence type *r*, relative to *equilocal* residence, are

$$\text{log}\left(\frac{{{\pi }}_{r,c}}{{{\pi }}_{e,c}}\right)= {\varvec{x}}_{\varvec{c}}^{\varvec{{\prime }}}{\varvec{\delta }}_{\varvec{r}}+{W}_{r,l\left(c\right)}+{Y}_{{r,b}_{m}\left(c\right)}+{Z}_{r,{b}_{f}\left(c\right)}$$

where ***x***_***c***_ is a vector of covariates for couple *c* (the same covariates for each residence type *r*); **δ**_*r*_ is a vector of regression parameters for residence type *r*; *W*_*r*_ is a family-level varying intercept for residence type *r*; and *Y*_*r*_ and *Z*_*r*_ are respectively male and female birthplace-level varying intercepts for residence type *r*.

The covariates ***x***_***c***_ in our baseline model for postmarital residence include an additive term for the *subject’s birthyear*, used to adjust for secular trends, additive terms for *generation* and *region*, and their pairwise interaction. The full-factorial model for *generation* and *region* allows each of the four combinations of these variables to have a unique effect on each residence type *r*. Similar to the migration model, elaborations of the baseline model include either additive terms for the *size* of the settlement of the couple’s place of postmarital residence or additive terms for the subject’s *genetic ancestry*. Couples whose postmarital residence type could not be determined due to missing information (*n* = 314 of 1173) were excluded from the analysis.

### Computational Method

We turned to computational Bayesian methods to fit the migration and postmarital residence models for the following reasons: (1) the need to adjust for many family lineage and birthplace effects using varying intercepts, some of which are likely to have relatively weak statistical support because they are shared by only a few individuals; (2) the need to incorporate different forms of censoring of migration distances; and (3) the fact that these computational demands are combined in the present study with non-standard outcome distributions (hurdle, multi-category). The Stan modeling language (Stan 2.2.1; Stan Development Team 2020), a computational Bayesian platform implementing Hamiltonian Monte Carlo methods (Monnahan et al., 2017), provides efficient computation as well as a high degree of flexibility to meet unique modeling needs. Statistical inferences using this and other related approaches are based on computer-generated stochastic samples from the joint density of the model parameters, given the observations (Jackman, 2009). Graphical displays of these *posterior densities* depict the sign, magnitude, and uncertainty of model estimates, among other properties.

Basic templates in the Stan language for the hurdle and multi-logit models were provided by McElreath and Koster (2014) and Koster and McElreath (2017), and we modified these as needed for the present work. We incorporated censoring of migration distances by integrating their probability densities over the ranges given by the minimum and maximum distances known for each censored case, using techniques described in the Stan User’s Guide (Stan Development Team 2020; see “Genetic Data” above).

In choosing prior parameter densities, we attempted to balance the desire for full exploration of the parameter space with regularization, which aims to prevent unstable or nonsensical model estimates (Gelman et al., 2017). In the migration model, the vector of regression coefficients for the log-odds of migration, **β**, has a multivariate Gaussian prior with mean 0 and variance/covariance matrix **Σ**_**β**_, the latter having a second-level prior based on a Cholesky factorization (details are in the Stan User’s Guide, Sect. 1.13, Stan Development Team 2020; see also McElreath & Koster 2014). The family and birthplace varying intercepts, *S* and *T*, have independent Gaussian priors with mean 0 and standard deviations σ_*S*_ and σ_*T*_, the latter having second-level half-Cauchy priors with location 0 and scale 1. The regression coefficients for log-distance, ***γ***, have a multivariate Gaussian prior in which the mean vector has a first element equal to log(100), used as a prior baseline, and all subsequent elements are equal to 0. The variance/covariance matrix **Σ**_**γ**_, and all prior specifications related to *U*, *V* and *E*, are treated analogously to the model for the log-odds of migration.

The postmarital residence model, incorporating sub-models for neolocal, matrilocal and patrilocal residence, has a multivariate flavor reflected in our prior specifications. The concatenated vector of regression coefficients, **δ’** = (**δ’**_neolocal_, **δ’**_matrilocal_, **δ’**_patrilocal_), has a multivariate Gaussian prior with mean 0 and variance/covariance matrix **Σ**_**δ**_, treated analogously to **Σ**_**β**_ in the migration model. The concatenated form of **δ** accommodates the possibility that covariate effects may be correlated across residence types. Based on the same reasoning, the priors for family and birthplace varying intercepts in the postmarital residence model also apply to concatenated forms: ***W*** = (*W*_neolocal_, *W*_matrilocal_, *W*_patrilocal_) has a multivariate Gaussian prior with mean 0 and variance/covariance matrix **Σ**_***W***_, the latter treated analogously to **Σ**_**β**_ in the migration model. This specification for W accommodates the possibility that family-level varying intercepts may be correlated across residence types. Concatenated male and female birthplace-level varying intercepts, ***Y*** and ***Z***, are treated in the same way.

The model estimates presented here derive from 1,500 samples of each parameter from each of 4 independent chains (6,000 total samples), after 6,500 samples per chain as a warmup. We present example trace plots from four chains in the ESM (Figure S4) to illustrate Stan’s rapid convergence and efficient mixing.

Expected log predictive densities for model comparison were calculated using Pareto smoothed importance-sampling leave-one-out cross-validation (PSIS-LOO) as implemented in the R package loo (Vehtari et al., 2017). Scripts for processing the demographic data, running, and plotting the demographic models are available at https://github.com/reynolds-lab/HumNat2023_analysis.

## Results

### Demographic Data Summary

To better understand the changing demographic patterns in this region of southern Africa, we collected detailed demographic interviews and DNA samples from 410 individuals living in two rural regions of the Northern and Western Cape Provinces of South Africa (Fig. 1a): the Cederberg (*N* = 204), and the Richtersveld (*N* = 206). All individuals included in the study were adults (age range, 18–94 years; mean, 55 years; 253 women, 157 men). We collected information about the age, birthplace, spoken languages, and self-identified ethnicity of the participants, as well as of their parents and grandparents. Our final dataset represents the birthplaces of 2870 individuals living in these areas over the past three generations.

In the Cederberg, the majority (92%) of individuals included in our analysis self-identify as Coloured. We sampled 70 men and 134 women born in 16 locations across the region (Fig. 1c). After including parental and grandparental birthplaces, this sample represents 111 towns and farmsteads across the region. The majority of individuals (76%) living in the Richtersveld are Nama (an ethnic group who were historically Khoekhoe pastoralists), with the remainder identifying as Coloured. We sampled 87 men and 119 women born in seven locations across the region (Fig. 1b). Including parental and grandparental birthplaces, this sample represents 79 small towns and farmsteads across the region.

Intergenerational migration is characterized by comparing an individual’s birthplace with one or both of their parents’ birthplaces. In our dataset, there are two observations for each participant (a participant’s birthplace versus their mother’s or father’s), as well as two observations for each parent of a participant (each parent’s birthplace versus their mother’s or father’s). Migration data were missing for ~ 27% of our dataset, leaving a total of 1717 possible migration events for analysis. Migration status was available more often for parents than grandparents (Table S19: Migration Status × Relation to Proband), apparently due to better knowledge among probands of parents’ birthplaces than of grandparents’ birthplaces. Migration status was available more often for probands’ mothers and maternal grandparents than (comparatively) for probands’ fathers and paternal grandparents. It could be surmised that a proband’s knowledge of a grandparent’s birthplace would be negatively impacted by migration of a parent. Our sample supports this conjecture (Table S20: Migration Status of Grandparents Conditional on Parent’s Migration Status), with grandparents’ migration status available relatively more often when a parent did not migrate.

**Fig. 2:**
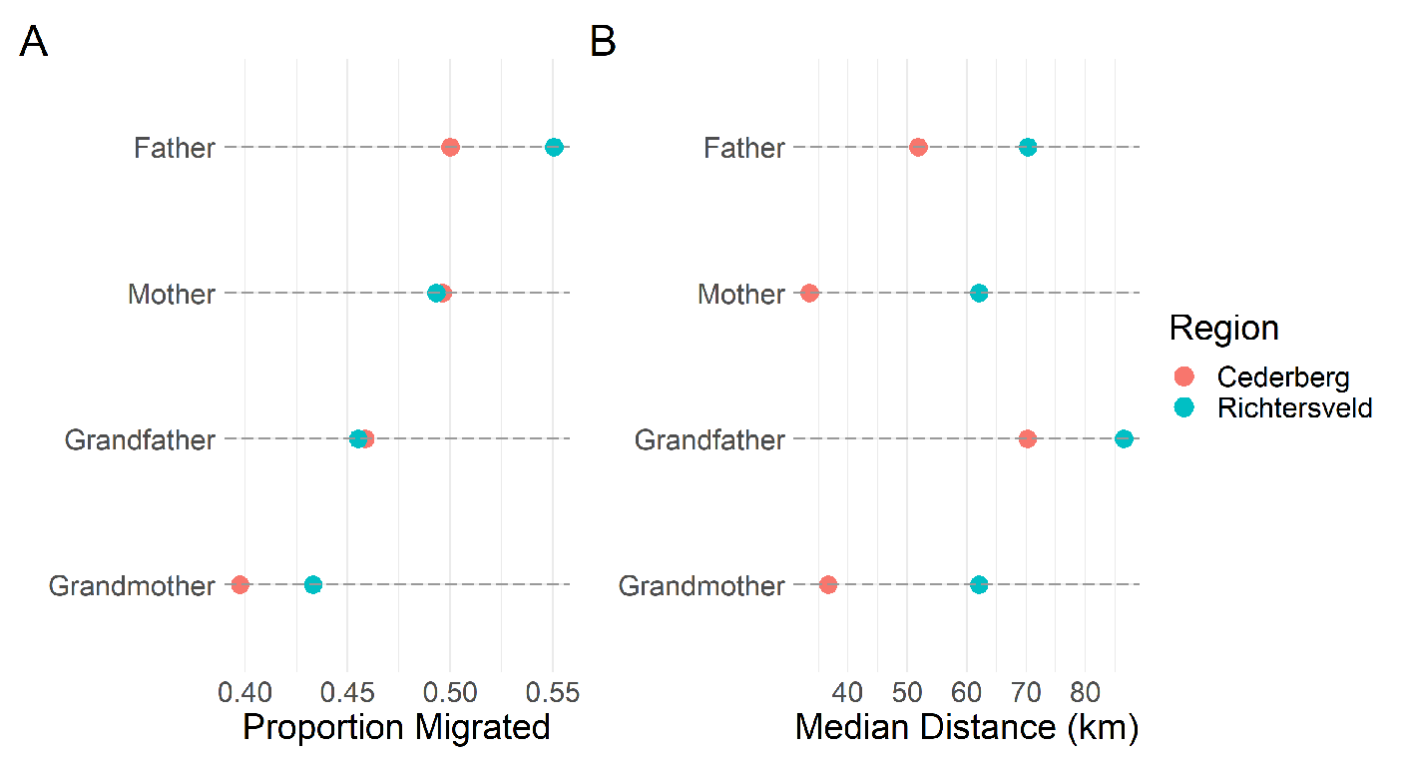
Observed proportions of individuals in the sample who migrated from their natal location (A) and median migration distances (B) by region, generation, and gender. Larger proportions of the parental generation (mothers and fathers) migrated, compared to the grandparental generation in both study regions, and grandmothers had the smallest proportion of migrants. For all categories, individuals in the Richtersveld migrated longer distances than individuals in the Cederberg. Men migrated longer distances than women in both study regions

Just under half (47%) of individuals in our dataset of complete cases migrated, but this varied by gender, region, and generation (Table S1; Fig. 2a). Specifically, a higher percentage of parents migrated than grandparents and a higher percentage of men migrated than women. For individuals who migrated, the median distance was 60 km (IQR = 106 km). This also varied substantially by gender, region, and generation (Fig. 2b). In both the Cederberg and the Richtersveld, women moved shorter distances than men and the younger generation moved shorter distances than the older generation. People in the Cederberg moved shorter distances on average than people in the Richtersveld.

The postmarital residence patterns in our dataset are shown in Table S2. Locality data were missing in ~ 27% of our dataset, leaving 859 observations of postmarital residence patterns for analysis. Locality status was available more often for parents than for grandparents (Table S21), and more often for maternal grandparents than for paternal grandparents. The locality status of grandparents was recovered most readily when parents settled equilocally (Table S22), and relatively less readily for neolocal, matrilocal, and patrilocal parents.

The most common pattern of postmarital residence among the complete cases is an individual with the same birthplace as both parents (equilocality). Among the remainder, 55% had birthplaces different from both parents (neolocality), 25% shared a birthplace with their mother but not their father (matrilocality), and 19% shared a birthplace with their father but not their mother (patrilocality). The median distance between an individual’s birthplace and their father’s birthplace was 68 km (IQR = 137 km) and the median distance between an individual’s birthplace and their mother’s birthplace was 49 km (IQR = 58 km), excluding individuals born in the same place as both parents.

### Genetic Data Summary

In order to better understand the relationship between genetic ancestry, migration, and postmarital residence, we collected genetic data from participants. We successfully genotyped 317 study participants for more than 1.5 million genome-wide SNPs on either the Illumina MEGA or H3Africa arrays (*N* = 152 Cederberg, H3Africa; *N* = 165 Richtersveld, MEGA). We used a global ancestry analysis to estimate the proportion of each participant’s genetic ancestry coming from recent Khoe-San, West/Central African, or Eurasian ancestors. This analysis, implemented in the ADMIXTURE program (Alexander et al., 2009), is a model-based clustering technique that takes genetic data from individuals (in our case, single nucleotide polymorphisms) as input and models those individuals as having a proportion of their genetic markers belonging to one or more of the clusters. For example, an individual could have 40% of their markers belonging to cluster A and the other 60% belonging to cluster B, assuming only two clusters. This approach can be used in recently admixed populations (those that have ancestors from two or more previously isolated groups) to infer the proportions of ancestry an individual has from each source population. Admixture analyses include not only the admixed population of interest but also potential source populations, or populations closely related to the possible source populations. In our study region, historical records suggest the indigenous Khoe-San groups in southern Africa interacted primarily with European colonists. Cape Town, however, had a large presence of enslaved individuals from Indonesia, South Asia and elsewhere from sub-Saharan Africa, so we included comparative populations from all five of the potential source populations when estimating the admixture proportions in our participants.

**Fig. 3:**
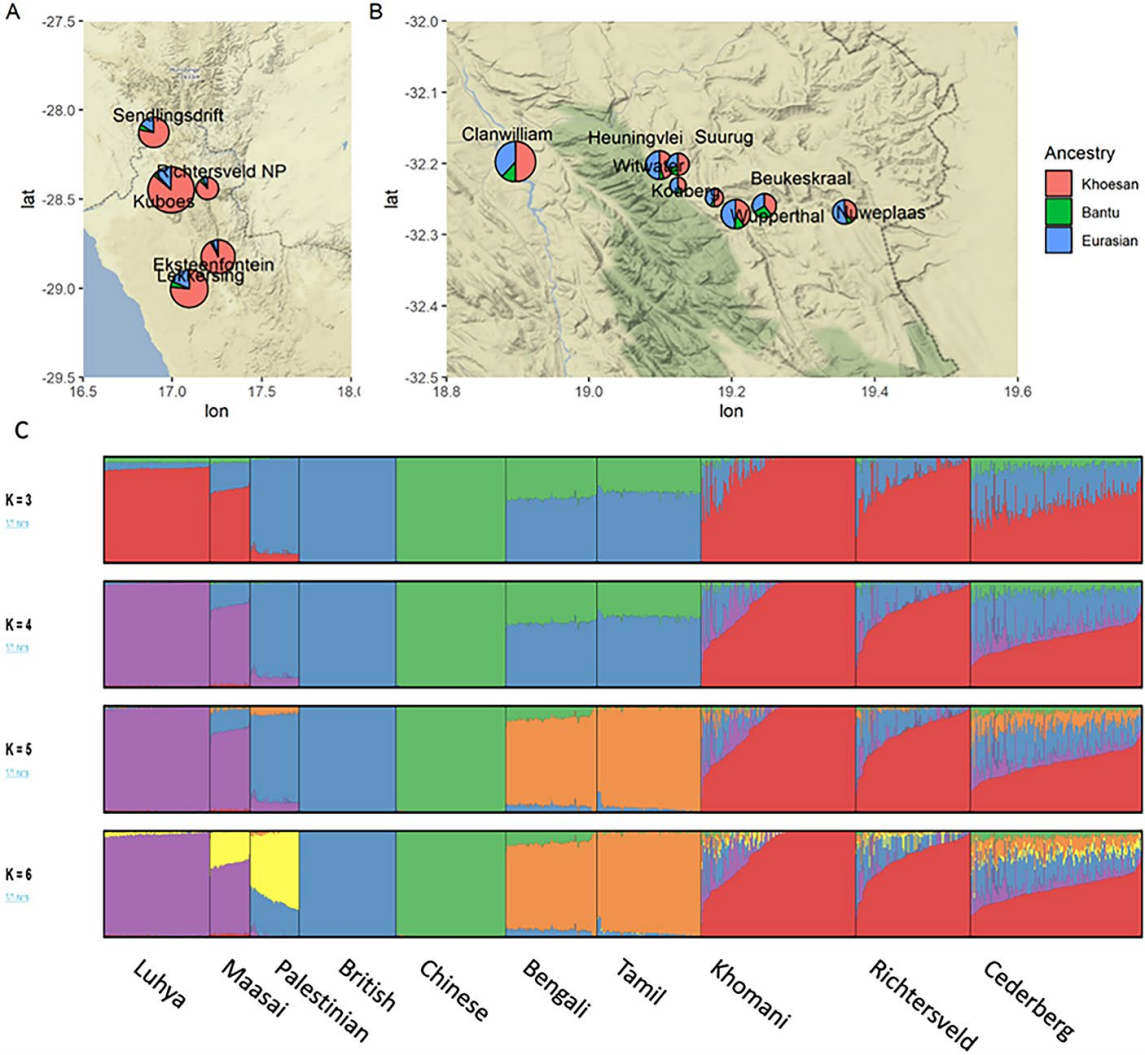
Average ancestry proportions of the three major genetic ancestries by sampling site in the Richtersveld (A) and the Cederberg (B). The Richtersveld has much higher Khoe-San ancestry than the Cederberg, and much lower West African ancestry on average. Only sampling sites with > 5 individuals are included in the plot. (C) ADMIXTURE analysis of the study populations assumed *K* = 3–6 source populations, with a comparative dataset incorporating samples from the 1000 Genomes Project and the Human Genome Diversity Project. Each vertical band within a population group represents a single individual, and the various colors represent the proportion of that individual’s genome inferred to come from a putative source population. Study participants’ genomes, shown in the two right-most panels for each value of *K*, tend to be dominated by the red cluster, corresponding to an inferred Khoe-San ancestry, for *K* = 4:6. Lesser proportions belong to the purple cluster (inferred West African ancestry), the blue cluster (inferred European ancestry), the green cluster (inferred East Asian ancestry), and the orange cluster (inferred South Asian ancestry)

Global admixture analysis (Fig. 3) shows a high level of Khoe-San ancestry in both the Richtersveld and the Cederberg. Genetic contributions from Eurasian and West African groups are greater in the Cederberg than in the Richtersveld. Following common practice, we present the ADMIXTURE results using various numbers of clusters (*k* = 3:6) to show the different population structure that emerges as the number of clusters changes. At *k* = 3 we can see three primary ancestry clusters: African, European, and East Asian. At *k* = 4 we see a division of the African cluster into a West African cluster and a Khoe-San cluster. At *k* = 5 we see the South Asian populations form their own ancestry cluster. At *k* = 6 we see a component most common in Palestinians, but to a lesser extent in the Maasai from Kenya and the Nama from the Richtersveld. Because East and South Asian ancestry is seemingly a small fraction of the overall ancestry in our study populations (particularly in the Richtersveld), we used three ancestry categories (Khoe-San, West African, and Eurasian) for subsequent modeling. Our study design permitted only the genetic sampling of the study participant (the “proband”); therefore, the genetic ancestry of the participant is used as a proxy for the ancestry of each family member. We hypothesized that higher levels of genetic ancestry from one population might be used as a crude proxy for more cultural interaction and provide additional explanatory power when estimating migration or locality patterns.

### How does Migration Differ Between the Cederberg and Richtersveld, and Across Generations?

**Fig. 4:**
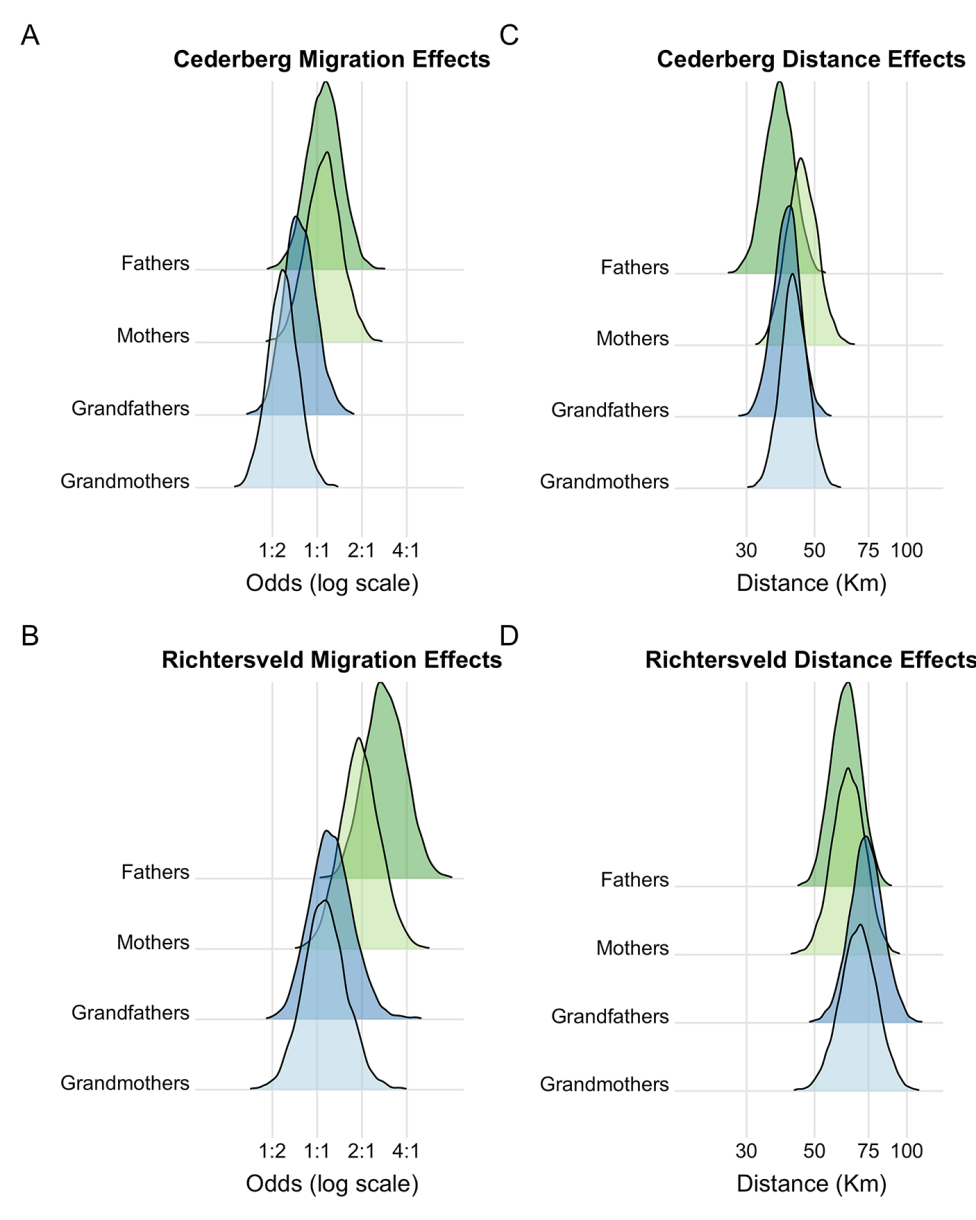
Posterior densities of the odds of migration and distance migrated, by sex and generation, for the Cederberg (A and C) and the Richtersveld (B and D) based on the migration model. The migration model adjusts for subject birthyear, and the effects shown here refer to parents and grandparents of subjects born in the sample median birthyear, 1957. Family members in the Richtersveld exhibited greater odds of migration and longer migration distances than those in the Cederberg. The parental generation had greater odds of migration than the grandparental generation, and these differences were more pronounced in the Richtersveld. For visualization purposes, the posterior samples used to create the densities were marginalized over varying intercepts for birthplaces, with the relative frequencies of birthplaces in each combination of region, generation and sex as weights

We fitted a hierarchical model (“Modeling Intergenerational Migration,” above) to investigate the relationship between migration and the following combined factors: region (Cederberg or Richtersveld), generation (parental or grandparental), and gender. Parents and grandparents born in the Richtersveld were more likely to have migrated, and migrated longer distances on average, than their counterparts in the Cederberg, as shown by comparisons across regions in Fig. 4. Generational differences in the odds of migration—with parents more likely to have migrated than grandparents—are also evident by comparing the blue- and green-shaded densities on the left-hand side of Fig. 4. Grandmothers in the Cederberg stand out as being the least likely group to have migrated (Tables S5, S6). We adjusted for the subject’s birth year in the migration model because of the wide age range across the sample. We found statistically well-supported, positive coefficients for this variable for both the odds of migration and migration distance, suggesting a secular trend: the parents and grandparents of younger subjects were seemingly more likely to migrate, and migrated longer distances, than those of older subjects.

We included varying intercepts (“random effects”) for birthplaces and families to learn about the magnitudes of these contextual effects relative to the baseline effects of region, generation and gender. Some birthplaces show strong “leave” or “stay” effects on migration, (Figure S1A; Table S16) and highly variable effects on migration distances (Figure S1C). The propensities of families to migrate across generations and their migration distances were not as variable, with most family-effect densities centered on zero (Figure S1B, S1D).

### How do Postmarital Residence Patterns Differ Between the Cederberg and Richtersveld, and Across Generations?

We fitted a hierarchical model to investigate the relationship between postmarital residence and the combined factors, region (Cederberg or Richtersveld) and generation (parental or grandparental). Under our data collection methods, postmarital residence form (e.g., neolocal) was determined by comparing an individual’s birthplace with the birthplaces of his or her parents (see “Demographic Data” for full details).

**Fig. 5:**
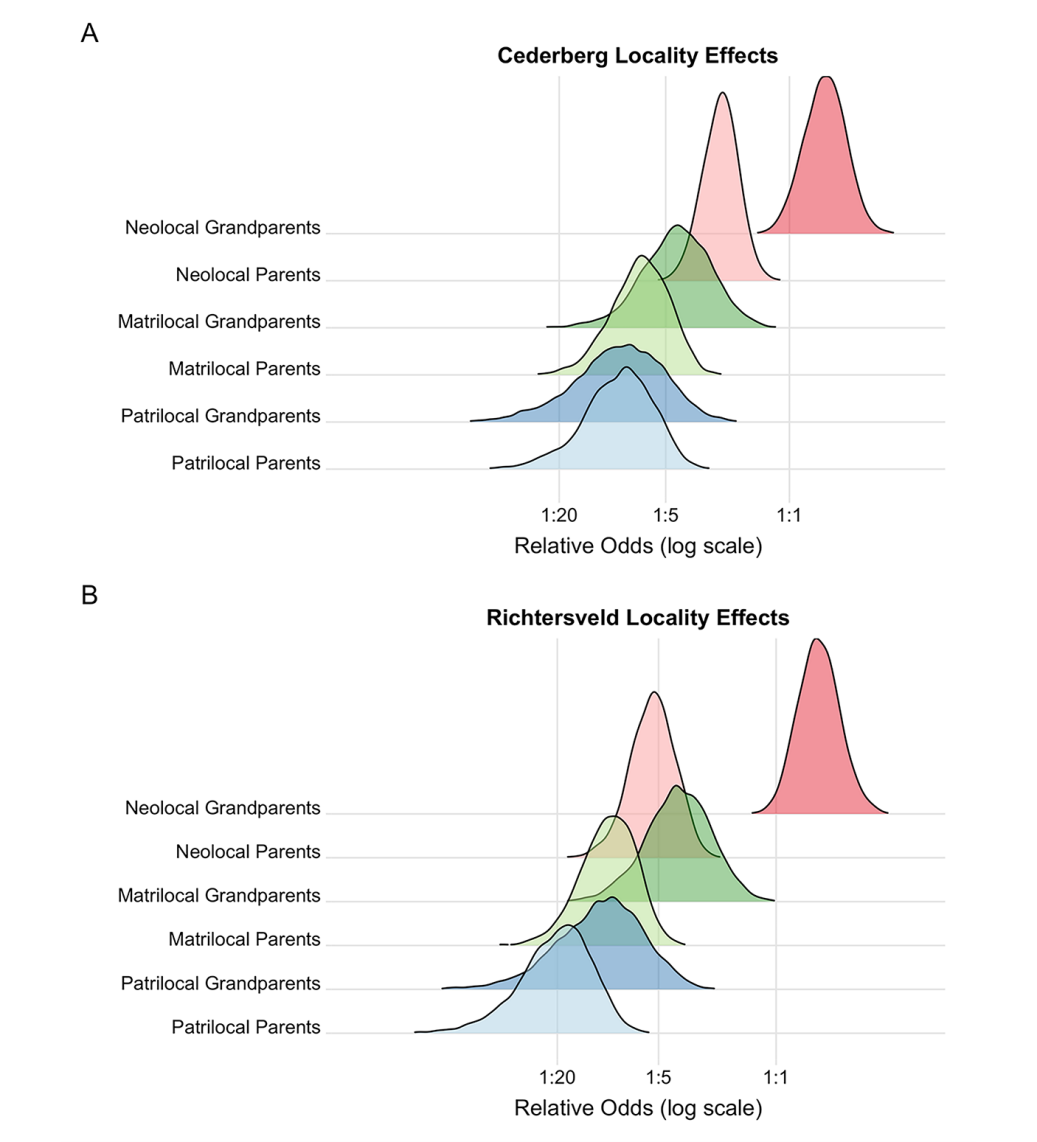
Posterior densities of the odds of neolocal, matrilocal and patrilocal postmarital residence, relative to equilocal residence, for the Cederberg (A) and the Richtersveld (B) based on the postmarital residence model. The results are ordered by generation within locality as our primary focus is on intergenerational change in locality status. Neolocality and matrilocality were more common (relative to equilocality) in Cederberg parents, compared to their counterparts in the Richtersveld. As for the migration model, the posterior samples used to create the densities were marginalized over varying intercepts for birthplaces

Grandparents in both regions were highly neolocal, favoring this residence mode even over equilocality (Fig. 5). In the parental generation, parents in the Cederberg were more likely to reside neolocally (relative to equilocally) than their counterparts in the Richtersveld. Viewing the densities upward from the bottom in the Richtersveld, the position of the dark green density suggests that matrilocality was common among grandparents of this region. In both the Cederberg and the Richtersveld, grandparental densities are horizontally separated (darker shading), suggesting a rank ordering of postmarital residence preferences. In contrast, parental densities show more overlap (lighter shading), suggesting that parents in both regions showed similar preferences for the other forms of postmarital residence compared with equilocality. Finally, patrilocality was the least common residence pattern across generations and regions. Adjustment for subject birth year in the postmarital residence model produced a statistically well-supported, positive coefficient for the neolocal sub-model, suggesting a secular trend: parents and grandparents of younger subjects were more likely to reside neolocally than those of older subjects.

The model structure is more complex relative to the migration models (described above, “Methods”); the model for postmarital residence incorporates three sub-models (for neolocality, matrilocality, and patrilocality relative to equilocality) and the intercept for a birthplace or family differs in each. Further, both members of a married couple contribute a varying intercept for their respective birthplaces. Similar to the migration model, birthplace effects on postmarital residence can be profound, increasing or decreasing the relative odds that a couple chose a given postmarital residence pattern by an order of magnitude (Figure S2; Table S17). In contrast to the birthplace effects, family effects on postmarital residence are seemingly minor.

### Genetic Ancestry and Settlement Size as Predictors of Migration and Postmarital Residence Patterns

Since birthplace effects on migration probability and postmarital locality can be quite strong, we asked whether birthplace size or, alternatively, genetic ancestry offered additional explanatory power. We extended our models to include either a categorical predictor encoding the settlement size of an individual’s birthplace or the genetic ancestry ratios of the proband to determine if these variables would further explain migration and locality above and beyond the baseline model. After fitting the base model and the two extended models, we performed model comparisons using an approximate cross-validation technique (Vehtari et al., 2017). Of the models we considered, the extended model incorporating settlement size had the highest support for both outcomes: migration and postmarital residence (Tables S3, S4). We first interpret effects of the preferred model, including settlement size, and subsequently the effects of the model including genetic ancestry.

We included varying intercepts (“random effects”) for families and birthplaces in the base model to acknowledge hierarchical structure (“Modeling Intergenerational Migration,” above) in the dataset and adjust for unmeasured contextual effects associated with these groupings. The estimated standard deviations of these effects, as well as the effects themselves, can be informative about their importance as sources of variation. The covariate settlement size arguably operationalizes the varying birthplace effect and may supersede it if settlement size is a strong predictor. A reduction in the standard deviation of birthplace effects would indicate if this were the case. Because many individuals were born on farms or in small settlements without officially recorded census sizes, settlement size was coded as a categorical variable (city, town, farm, and unknown). Among individuals who migrated, those born in towns or on farms tended to migrate shorter distances than those born in cities (Table S10). Comparing the base model to the model including settlement size (Table S8), we see a decrease in the birthplace standard deviation, suggesting that settlement size explains in part why individuals with different birthplaces migrated at different rates. An analogous decrease was not detected in the postmarital residence model.

Genetic ancestry was quantified by two ratios, the proportion of Khoe-San ancestry relative to Eurasian ancestry and, similarly, for West/Central African relative to Eurasian, as estimated by ADMIXTURE at *k* = 3. None of the ancestry coefficients in the migration or postmarital residence models could be statistically distinguished from 0, suggesting that the ancestry covariates provide very little additional information about these demographic outcomes (Table S11). The ancestry covariates could be said to operationalize varying family effects just as settlement size did for birthplace effects. However, no reductions were seen in the family standard deviations for either the migration or postmarital residence model, suggesting that the addition of genetic ancestry estimates provided no additional explanatory power to our models of migration or postmarital residence.

## Discussion

European colonialism had a massive impact on the cultural and social systems of Indigenous peoples around the world. Through multiple means, colonial forces displaced worldviews, languages, histories, and traditions. The interaction of European colonial powers and indigenous groups has varied across time and space; however, common themes emerge as colonial governments sought to obtain and control land masses. Settler colonialism, as this has come to be called, typically employs racial classification to variably incorporate, disenfranchise, or eliminate indigenous groups to solidify colonial control of the land (Wolfe, 2006). Historically this process often involved the conversion of indigenous groups to the colonial mindset through the work of missionaries (Allen, 2013; Comaroff & Comaroff, 1986; Merrill, 1993). This process has been called acculturation, whereby over time indigenous communities assimilate into the dominant culture and the memory of their cultural traditions is lost. Despite the inroads of dominant colonial cultures, many aboriginal cultural practices persist in contemporary indigenous and creole groups around the world. It is perhaps more accurate to describe this process, particularly in the South African context, as one of creolization, where a new culture is formed at the meeting place of formerly distinct cultures, taking practices from each and blending them together into something unique (Challis, 2012; Palmer, 2015).

The cultural transmission in the present study population must be placed in a historical context that accounts for the history of colonialism in South Africa and the power dynamics between indigenous peoples and the dominant colonial culture. In 1652 the Dutch East India Company (VOC) established a settlement at the Cape of Good Hope in what would become modern South Africa, beginning the colonial period in this region. For the first three decades of its existence, the Cape Colony remained very small, relying on trading with nearby indigenous Khoe-San groups for livestock to supply its ships (Guelke, 1976). The colony began expanding in 1679 as farmers occupied the nearby Cape Flats and the region around modern-day Stellenbosch. Settlers quickly spread out from the southwestern Cape, crossing the Berg River (which empties into the Atlantic Ocean near the modern town of St. Helena) by 1712 (Penn, 1986) and reaching the Cederberg mountains by the 1720s (Guelke, 1976). The increased need for labor in the Cape Colony was largely met by the Indian Ocean slave trade, although farmers who could not afford slaves turned to the indigenous Khoe-San population as a cheap labor source.

Since the beginning of the colonial period, the indigenous Khoe-San residents have experienced huge demographic changes. As the colonial frontier expanded, a group of people with parents from multiple ancestries emerged, referred to derogatorily as “Bastaards” (Penn, 2005). Their social standing in colonial society varied, but as the eighteenth century progressed, discrimination against Bastaard communities increased, resulting in many families fleeing to the northern and eastern frontiers of the Cape Colony. As these communities fled from the expanding colony, they encountered various indigenous Khoe-San groups, such as the Nama, and in many cases became integrated with them (Penn, 2005) or formed new communities (Edgar & Saunders, 1982; Nurse & Jenkins, 1975; Ross, 1975). Because of this history of colonial expansion, and the subsequent incorporation of Khoe-San and Khoe-San-descendant communities into colonial society, our expectation was to see the adoption of colonial cultural traits in our study populations. For example, the primary language in both the Cederberg and the Richtersveld is Afrikaans. However, our results suggest that more recent forces of apartheid policies and the wider integration into the wage labor market are likely the primary drivers of change in the cultural traits in our study.

### Demographic Consequences of Policies of Forced Sedentism and the Transition to a Wage Labor Market

Our findings based on the migration and postmarital residence models are consistent with a transition to a wage labor market over recent generations in the Cederberg and Richtersveld. The covariate *settlement size* appears to partly explain contextual effects of birthplaces, detected in baseline models, and improves model performance overall. Examining the settlements that have the greatest effects on migration or postmarital residence, we see that larger economic centers such as Cape Town and Clanwilliam act as migratory sinks, decreasing the odds that an individual born there will move away. This pattern of urbanization of Coloured individuals in the Western and Northern Cape over the course of the twentieth century has also been observed in other studies (Bekker & Cramer, 2003; Kok & Collinson, 2006; Zietsman, 1988) and could be the result of a combination of factors including increased economic opportunities in more urban locations as well as land degradation and property alienation in more rural locations.

In both the Cederberg and Richtersveld, we see a higher level of neolocality in the grandparental generation relative to equilocality, followed by a large decrease during the parental generation. This pattern may be partially attributable to better resolution of birthplaces in the parental generation. However, the decrease in neolocality in the parental generation coincides with the limitation of the movement of African individuals in South Africa in the twentieth century under apartheid. The median birth year of our participants is 1957. This is a key period in South African history as many of the policies limiting the movement of African individuals under apartheid were taking shape in the early twentieth century. Land reserves were established for “Non-White” (a term used in apartheid legislation; Epstein 2018; Posel, 2001) South Africans in areas away from white-owned farms (Barry, 2004). After apartheid became state policy in 1948, these land reserves were assigned to specific racial groups defined by the government, and it became increasingly difficult for people to move between these areas (Barry, 2004). Some participants in living memory recall repossession of their family farms in the Cederberg. In the Richtersveld, participants reported the repossession of communal grazing lands that were given over to white landowners during apartheid.

My family had a farm near Calvinia that was taken in 1945, during the apartheid. The family spread to Riebeck and other places. I was moved here. (a Cederberg resident)

When I was born, people could use the land freely. White people came into the district and took the land for themselves. We either were pushed out or worked for the white people. During apartheid, they arranged for non-whites to come live in the Richtersveld. (a Richtersveld resident)

Apartheid was the culmination of more than a century of policies of forced sedentism in the region. The British took control of the Cape Colony in 1806. In 1809 the colonial government passed a law that came to be known as the “Hottentot Code,” which required Khoe-San individuals to live sedentarily; consequently, many who were not already living at missions became servants to white landowners (Dooling, 2005). Further revisions to the law in 1812 required Khoe-San children who grew up on the farms to serve the landowner for 10 more years upon reaching adulthood, which quickly put an end to the nomadic lifeways of the Khoe-San peoples living within the colony. This was expanded on in the early twentieth century as the Mission Stations and Communal Reserves Act of 1909 and the Natives Land Act of 1913, among other legislation, served to draw formal boundaries around what had once been communal land across our study areas in the Cederberg and the Richtersveld, as well as dispossessed Khoe-San and Coloured landowners of their holdings (McLachlan, 2019). This legislation helped to change mission lands into labor reserves for commercial farming and mining interests. Subsequent apartheid-era legislation such as the Group Areas Act of 1950 further limited the movement of Coloured communities (Oakley, 2006), solidifying the sedentization started more than a century prior.

Further north in the Richtersveld, the Nama practiced transhumant pastoralism, following the seasonal vegetation patterns. As the Cape Colony expanded throughout the eighteenth century, the Nama retreated further north, absorbing other displaced Khoe-San groups from the Cape, as well as communities of escaped enslaved individuals and those of mixed ancestry (Carstens, 1966; Smith, 1995). Permanent colonial settlement in the Richtersveld did not begin until the early nineteenth century with the establishment of a number of Christian missions near the modern town of Pofadder in 1813 and at the farm of Leliefontein in 1816 (Rohde & Hoffman, 2008; Smith, 1995). A number of mining companies were established in the region throughout the 1850–1860s to exploit rich deposits of copper, diamonds, and other mineral resources in Okiep and elsewhere. At the same time, much of the communal land in this region outside of the missions was privatized, limiting the grazing lands for the Nama’s livestock (Barry, 2004). Following the Land Act of 1913, much of the Richtersveld became one of seven Coloured land reserves, and participation in the cash economy became more important to maintaining a household in this region (Oakley, 2006). Together these factors may have expedited the transition to sedentism in the Richtersveld. We observe a generational effect in our migration model in which parents were more likely to migrate than grandparents. These differences are more striking in the Richtersveld, consistent with the historical record suggesting that the transition may have begun later in this area since it is farther from the economic center of Cape Town.

Recent settlement patterns reflecting historical missionization in the Cederberg may also be detected in the migration model. Dutch colonists likely first expanded into the Cederberg Mountains in the early 1700s (Penn, 1986). Because of its rugged terrain, the Cederbergs served as an excellent hiding place for Khoe-San groups retreating from the expanding colony, as well as escaped enslaved individuals (Penn, 2005). There was much conflict between the Khoe-San and colonists living in the Cederberg during the 1730s, culminating in a frontier war in 1739 (Mitchell, 2002). This conflict largely ended organized Khoe-San resistance in the region (Penn, 2005), with many groups further retreating from the frontier, while others were absorbed into the settler culture as clients or forced labor (Mitchell, 2002). In the last quarter of the eighteenth century some Khoe-San people became landowners as well, gaining some level of freedom, but on colonial terms (Mitchell, 2002). The land rights of these Coloured farmers were never certain, as noted by our participants. Several participants in the Cederberg recalled family stories of grandparents or great-grandparents, who had their own farms, being forced off the land by white neighbors.

My great-great-grandparents had farms in the veld and the Boers came with guns and forced them off the land. They asked the Moravian church for permission to stay in the town. (a Cederberg resident)

Back then, some Coloured people lived on their own farms. They had fat-tailed sheep. My mother’s grandmother had her own farm. Their white neighbor told them, “You can’t live next to me, so I am taking your farm, it is now mine.” (a Cederberg resident)

The first missionary settlements were set up in the Cederberg during the 1730s and established a system to sedentize the migratory Khoe-San groups in the region. Under this mission system, the church owned all of the land and would parcel it out to individual families. Each family would have their own house and plot of land to farm (Granger, 1982). These plots would then be passed down through families, a tradition that carries on in some of our participants’ families today.

My father’s side were farmers. The church owned the land but oma and opa owned the herds. (a Cederberg resident)

This farm belonged to my mother’s family. They sold it to the church in Wupperthal. They work the farm but pay rent to the church. (a Cederberg resident)

This continued into the twentieth century since, under apartheid policy, Coloured individuals living in mission stations could still be granted title to rural lands (Bekker & Cramer, 2003). This form of intergenerational land tenure may be reflected in our migration model: the majority of birthplaces that reduce an individual’s propensity to migrate—yet are not economic centers—are former mission settlements.

### Persistence of Matrilocal Residence Patterns

In many African hunter-gatherer societies, marriage is not formalized by a wedding ceremony, but rather through bride-service (Hill et al., 2011; Howell, 1979; Walker et al., 2011; Wood & Marlowe, 2011). As part of this process, the newly married couple spend the first years of marriage living with the family of the wife, and then after the birth of one or more children they may choose to continue living with the wife’s family, move nearer the husband’s family, or live somewhere else entirely (although typically following kinship ties; Marlowe 2004). This multilocal postmarital residence pattern with bride-service is considered to be a feature of gender egalitarianism among hunter-gatherer societies (Dyble et al., 2015) and can result in a residence pattern favoring postmarital matrilocal residence.

Historic and ethnographic evidence from many Khoe-San groups in our study region suggest a preference for matrilocal residence patterns, wherein a married couple tends to live closer to the woman’s family following the birth of their first child and a more ambilocal pattern for subsequent births (Barnard, 1992; Heinz, 1994; Lee, 1984; Silberbauer, 1981; Widlok, 1999). Under this postmarital residence pattern, the expected demographic patterns are that men will move away from their natal community more often than women and they will move greater distances than women. The evidence, based on migration data, for the persistence of a preference for matrilocal residence patterns is suggestive but not definitive. Our empirical migration data fit into this pattern quite well, with men moving farther than women and more often in both the Cederberg and the Richtersveld. In the Cederberg, the empirical pattern appears to shift in the parental generation, where men and women have very similar odds of migration. However, these apparent gender differences are attenuated in our models that adjust for subject birth year, along with contextual effects of birthplaces and families. Model-based estimates of the odds of migration, as well as the distance migrated, are very similar for males and females.

Postmarital residence patterns, like languages and other cultural traits, are subject to change and ultimately loss as a result of intercultural contact and interaction. This is partially a function not only of the power dynamics between the interacting groups (where a group might shift to the language and customs of their more powerful neighbors for social or economic benefits), but also the socioeconomic ecology of the area of cultural contact (Brenzinger, 1998; Mufwene, 2002). Historically, many European populations as well as South African Bantu-speaking populations were patrilocal (Blaauboer & Mulder, 2010; Fortunato & Jordan, 2010; Marks et al., 2012). Given the power dynamics between European colonizers and indigenous populations around the world, one might expect the Khoe-San to have assimilated to the patrilocal residence pattern during the colonial period. This adoption of colonial cultural traits is seen in contemporary Khoe-San and Khoe-San-descendant communities in South Africa since the vast majority of individuals speak the colonial language of Afrikaans as their first and primary language (instead of a Khoe-San language), practice Christianity, and variably practice farming or wage labor.

However, patrilocal residence patterns, which would have been historically practiced by the Dutch/British settlers who admixed with our study populations, are the least common in our dataset. Ambilocality and flexibility of movement are hallmarks of contemporary foraging societies. Instead of a complete transition to the historic locality patterns that might have been imposed by Dutch and British colonial governments, we see a diversity of locality patterns in Khoe-San and Khoe-San-descendent communities in South Africa today, suggesting that, despite the ever-increasing influence of globalization and the cash economy (Greenfield, 2016), there is a persistence of these behaviors in contemporary communities.

## Conclusion

Overall, our results show a pattern of urbanization and modernization in our study areas, whereby individuals are moving more often and greater distances than in the past. The size of a settlement seems to be particularly important in determining if someone will migrate or not, with more economically important locations such as Cape Town and Clanwilliam acting as migratory sinks, decreasing the odds that someone born there will move away. We see a variety of postmarital residence patterns in our study areas, with equilocality being the most common, followed by neolocality, consistent with patterns seen in Northern Europe leading to the Industrial Revolution (Hajnal, 1965; Hartman, 2004). Interestingly, we see the persistence of matrilocal residence, particularly among the Nama of the Richtersveld, and a dearth of patrilocal residence in our study areas. This suggests that despite hundreds of years of colonial rule, more indigenous Khoe-San cultural traits may persist to a greater degree than previously realized. Because these patterns are stronger in the Richtersveld, it seems that there may be a geographic gradient of increased persistence of Khoe-San cultural traits as distance to the cultural and economic center of Cape Town increases. Racial divisions in the northern frontier seem to have been more fluid than elsewhere in the Cape Colony (Legassick, 1993; Maylam, 2001), possibly allowing for more mutual acculturation and the persistence of certain Khoe-San cultural traits. Future studies would benefit from further geographical sampling, collection of birth order information, and the inclusion of additional cultural traits (e.g., contemporary practice of bride-service) that persist in Khoe-San and Khoe-San-descendant communities today to explore the scale of this transition.

## Electronic Supplementary Material

Below is the link to the electronic supplementary material.

Supplementary Material 1

Supplementary Material 2
