# Supplementary material for: Persistence of Matrilocal Postmarital Residence Across Multiple Generations in Southern Africa
Source: Hum Nat. 2023 Jun 13;34(2):295–323. doi: 10.1007/s12110-023-09452-4 (PMC10353969; doi:10.1007/s12110-023-09452-4)
Supplement: Supplementary file 1 — Supplementary Material 1 [file 12110_2023_9452_MOESM1_ESM.pdf]

Electronic Supplementary Material (ESM) for

Persistence of Matrilocal Postmarital Residence across Multiple Generations in Southern Africa

Austin W. Reynolds, Mark N. Grote, Justin W. Myrick, Dana R. Al-Hindi, Rebecca L. Siford, Mira Mastoras, Marlo Möller, and Brenna M. Henn

Corresponding author: Austin_reynolds@baylor.edu

Human Nature 34(2), 2023, https://doi.org/10.1007/s12110-023-09452-4

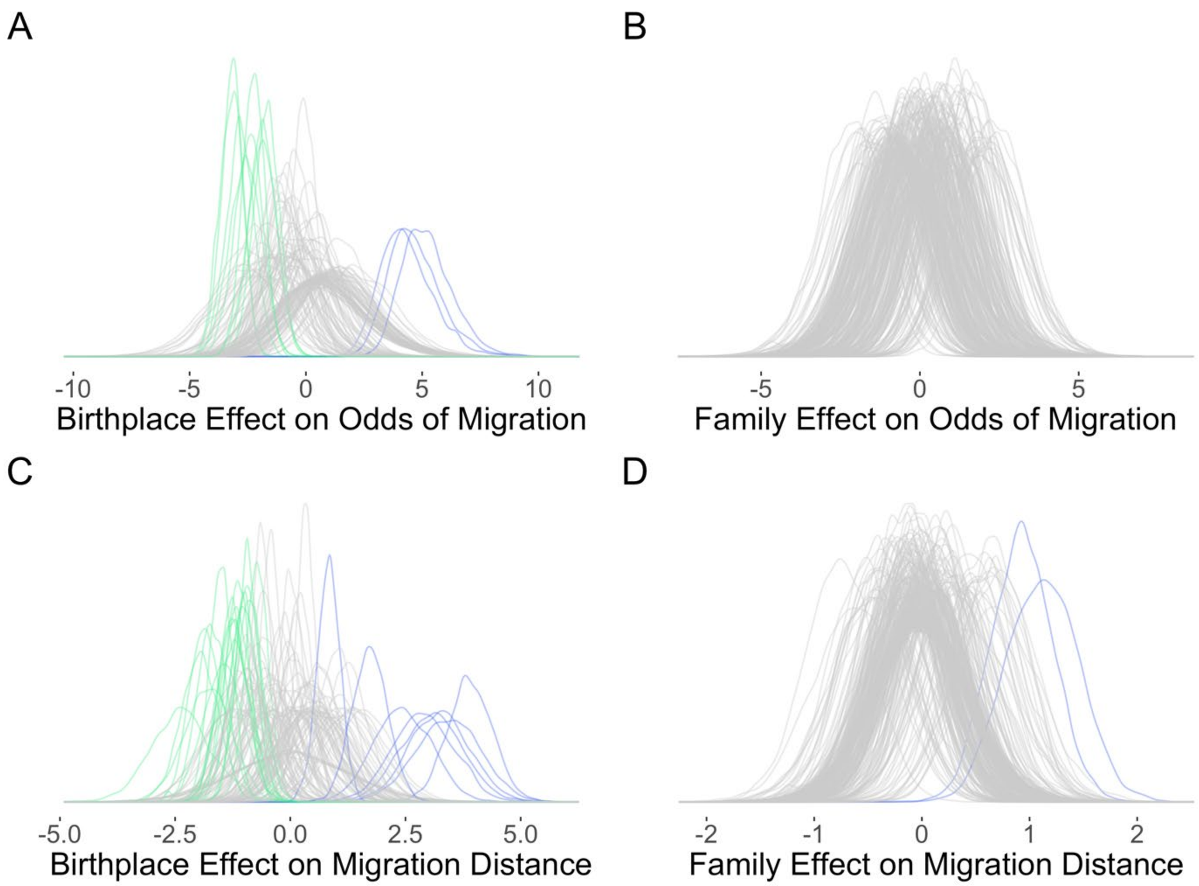

Figure S1: Posterior densities of varying intercepts for birthplaces and families, for the odds of migration (A, B) and migration distance (C, D). Birthplaces or families associated with unusually large odds of migration or longer migration distances relative to baselines are shown in blue, while those associated with unusually small odds or shorter distances are shown in green.

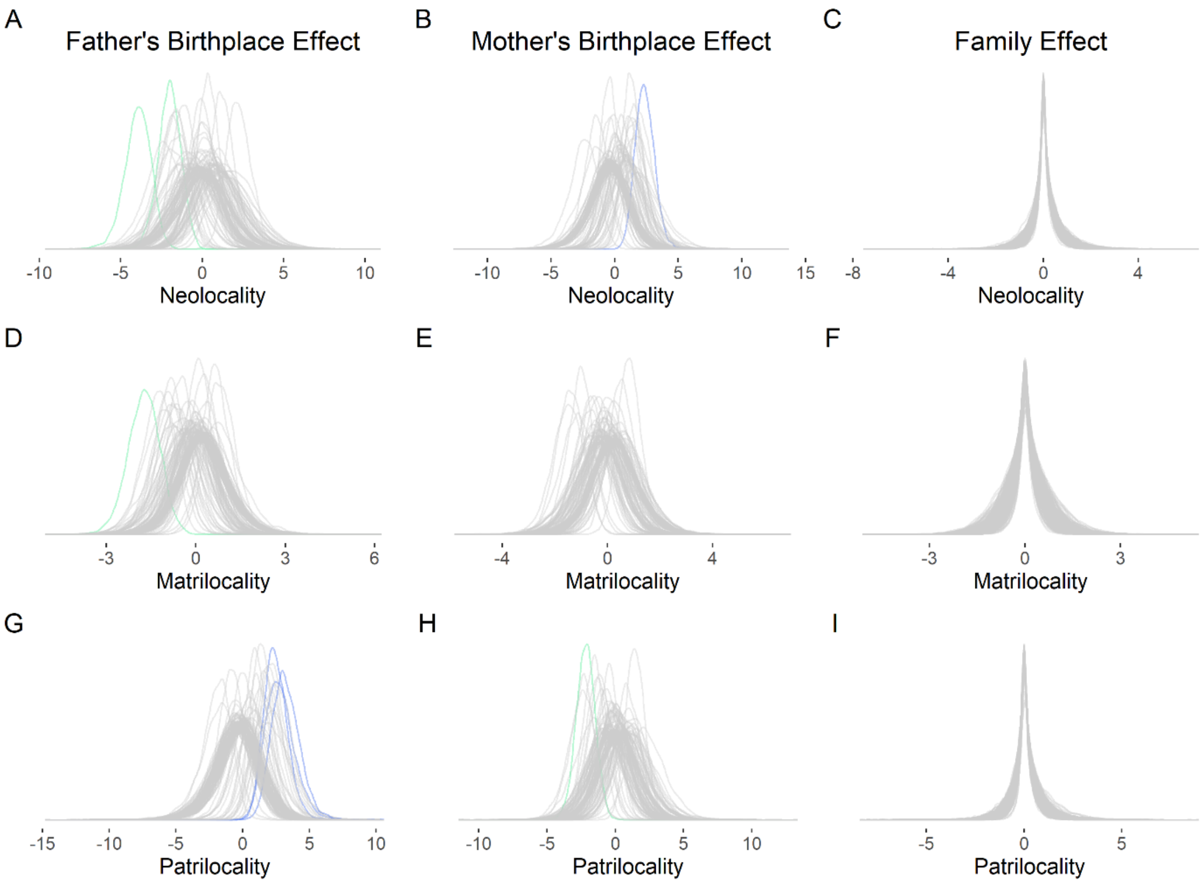

Figure S2: Posterior densities of varying intercepts for birthplaces and families, for the various locality patterns. Birthplaces or families associated with unusually large odds of neolocal, matrilocal, or patrilocal residence relative to equilocal are shown in blue, while those associated with unusually small odds are shown in green.

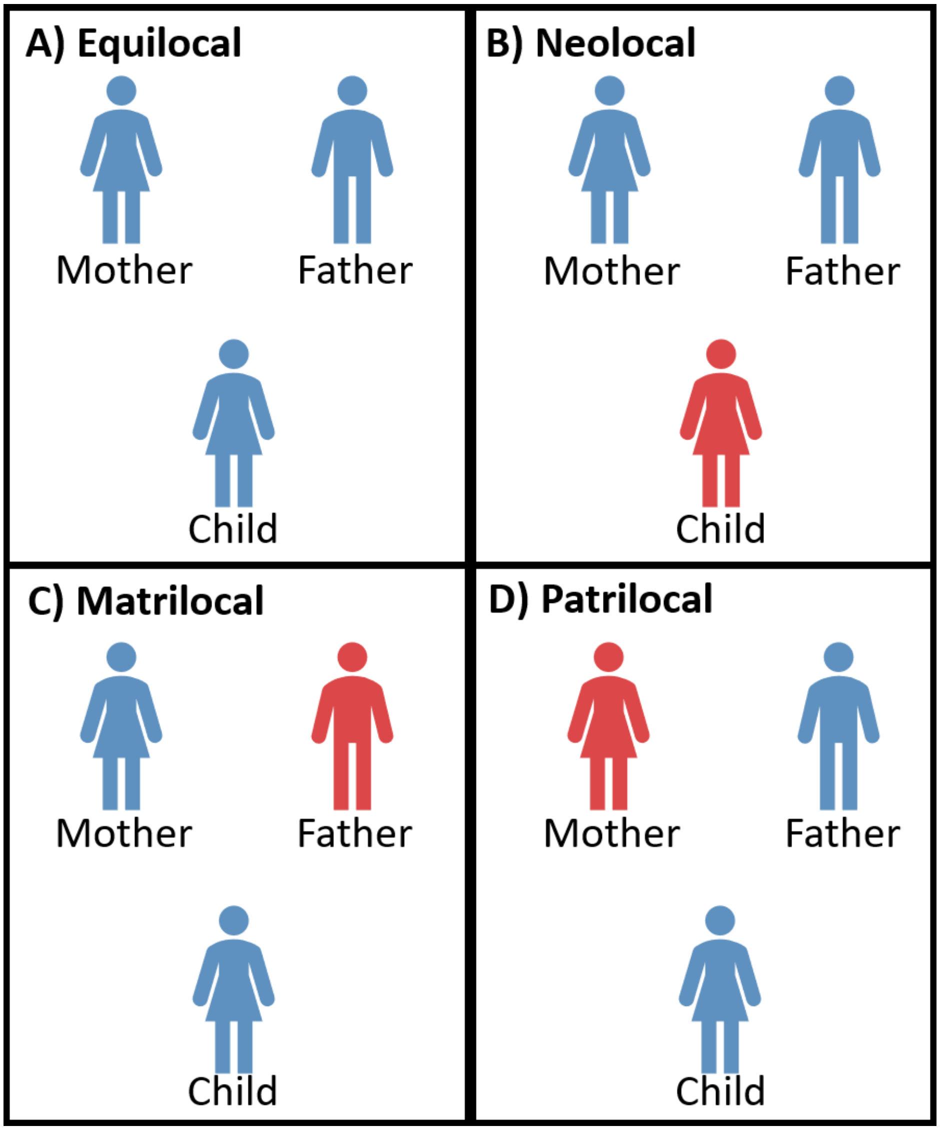

Figure S3: Visual explanation of the locality definitions used in the current study. Individuals sharing a birthplace in this figure are shaded blue and those with a different birthplace are shaded red. A) Equilocality is defined as a child having the same birthplace as both parents. B) Neolocality is defined as a child having a different birthplace from both parents. Importantly for neolocality, mother and father need not share a birthplace. C) Matrilocality is defined as a child sharing a birthplace with their mother but not their father. D) Patrilocality is defined as a child sharing a birthplace with their father but not their mother.

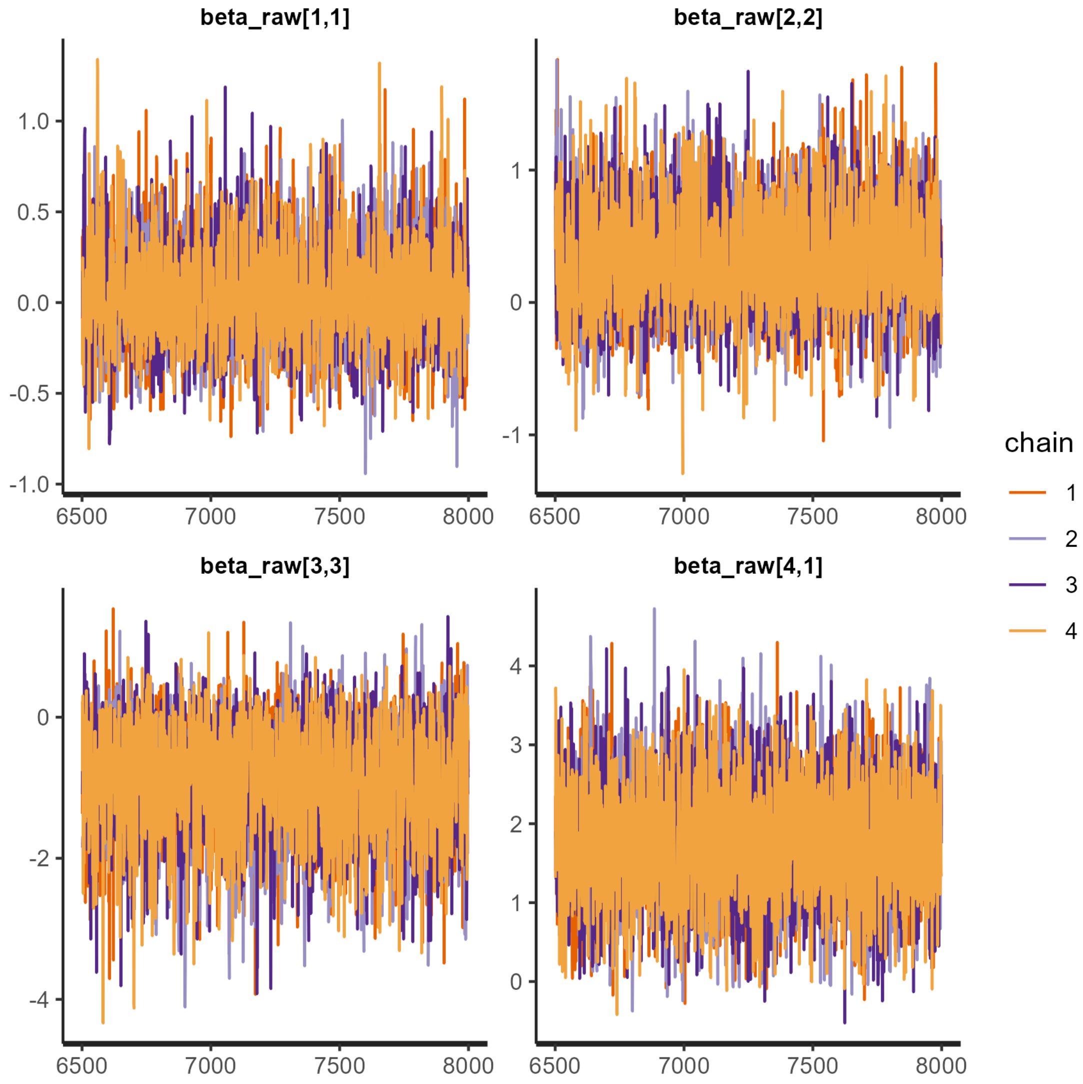

Figure S4: Representative trace plots from the locality model, demonstrating efficient mixing in all MCMC chains.
